# Recent Advances in Aptamer-Based Sensors for Sensitive Detection of Neurotransmitters

**DOI:** 10.3390/bios13040413

**Published:** 2023-03-23

**Authors:** Joon-Ha Park, Yun-Sik Eom, Tae-Hyung Kim

**Affiliations:** School of Integrative Engineering, Chung-Ang University, Seoul 06974, Republic of Korea; joonha95@cau.ac.kr (J.-H.P.); eys0521@cau.ac.kr (Y.-S.E.)

**Keywords:** aptamer, biomaterials, neurotransmitters

## Abstract

In recent years, there has been an increased demand for highly sensitive and selective biosensors for neurotransmitters, owing to advancements in science and technology. Real-time sensing is crucial for effective prevention of neurological and cardiovascular diseases. In this review, we summarise the latest progress in aptamer-based biosensor technology, which offers the aforementioned advantages. Our focus is on various biomaterials utilised to ensure the optimal performance and high selectivity of aptamer-based biosensors. Overall, this review aims to further aptamer-based biosensor technology.

## 1. Introduction

Neurotransmitters are molecules responsible for transmitting signals between nerve cells and play crucial roles in the central nervous system and the cardiovascular system [1,2]. Since most neurotransmitters activate receptors and continuously excite or inhibit neurons that have received signals, maintaining appropriate levels of neurotransmitters is essential [3,4,5,6]. Neurotransmitters can be divided into three major classes—amino acids, monoamines, and peptides. Glutamate, gamma-Aminobutyric acid (GABA), and glycine are members of the family of amino acid neurotransmitters and are involved in most nervous-system functions. Serotonin, histamine, dopamine, epinephrine, and norepinephrine play roles in the nervous system, especially in the brain, to regulate consciousness, cognition, attention, and emotions. Members of the peptides family, such as endorphins, act as natural analgesics [7,8,9,10]. However, inappropriate levels of neurotransmitters have been implicated as a cause of heart failure, cardiotoxicity, Parkinson’s disease, and Huntington’s disease [11,12,13,14]. Therefore, it is evident that quantifying neurotransmitters is necessary to monitor the effects of neurotransmitters and disease severity in real time. It has been reported that the development of ultra-sensitive neurotransmitter biosensors with sensitivity at the levels of ≤ nano units is meaningful for early disease diagnosis and monitoring [15,16,17,18,19]. In this context, the fusion of biosensors and biomaterials enhances the potential for sensing specific biomolecules [20,21,22,23,24,25]. In particular, biocompatible molecules, such as DNA- and RNA-based nucleic acids, are highly beneficial for the sensitive and selective detection of analytes. In addition, among the many nucleic acids, aptamers are recognised as noteworthy materials due to their valuable properties.

**Figure 1:**
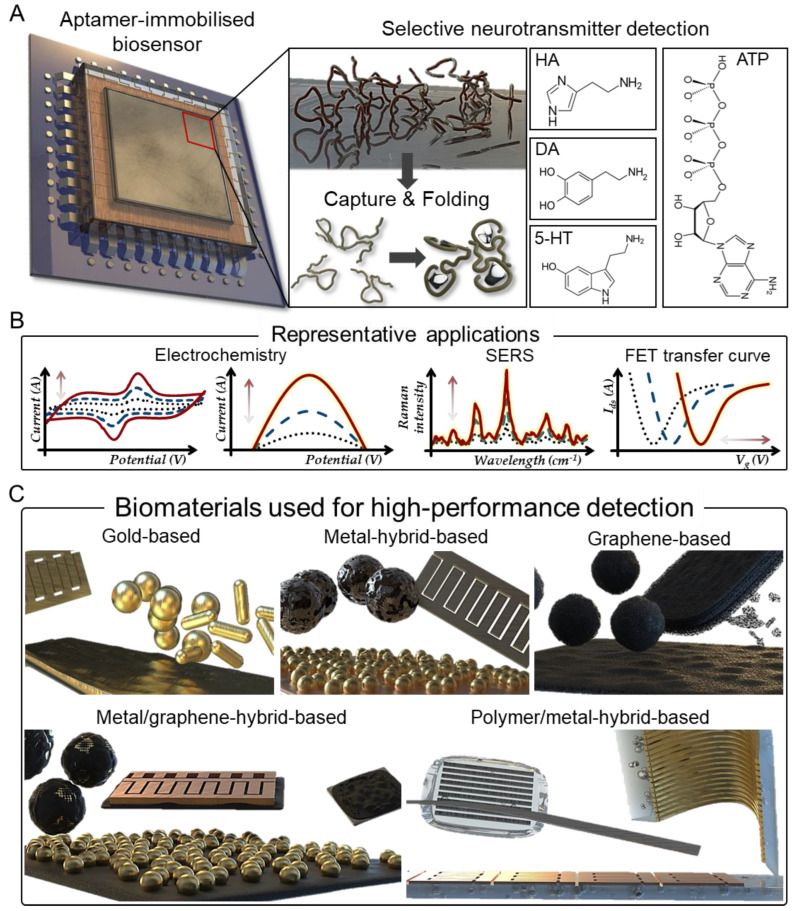
Aptamer biosensors for selective neurotransmitter detection. (**A**) Schematic indicating selective neurotransmitter detection with capture and folding process of aptamer. (**B**) Representative detection applications of aptamer-based biosensors. (**C**) Various forms of aptamer-based biosensors for high-performance detection applications.

Aptamers are peptides or oligo-nucleic acids with high sensitivity and selectivity for the detection of various types of analytes ranging from nucleotides, peptides, proteins, and small molecules to cells [26,27,28]. Aptamers consist of folded structures of single-stranded oligonucleotides that are typically 20–60 bases in length and are selected in vitro through the systematic evolution of the ligand exponential enrichment (SELEX) process [29,30,31]. Aptamers designed in this way offer distinct advantages over some unique antibodies in analyte analysis [32,33]. Small nucleic acid aptamers exhibit high structural stability under harsh conditions and have suitable structures to interact with the corresponding analytes [34,35]. Aptamers can be adapted to a variety of assays by introducing several modifications to their portion, including variable stem and loop regions that bind to specific target molecular pockets (Figure 1A) [36]. Furthermore, studies have reported that that aptamers can target cell-surface transmembrane proteins, as they are functional for the native forms of analytes in living cells [37,38]. Therefore, in addition to these advantages, integrating aptamers with diverse biomaterials generates a synergistic effect that can produce aptamer/biomaterial-based sensors capable of efficient analyte analysis and monitoring applications (Figure 1B).

Aptamer sensors based on various new biomaterials have been developed. For example, an aptamer sensor based on a gold material can be freely immobilised using various chemical conjugations [39,40,41,42,43]. In addition, gold has active electron-transfer effect and can amplify the electrochemical signal by the folding of methylene blue conjugated aptamers [44,45,46]. Hybrid forms using different metals can amplify the signal for the aptamer by enhancing the conductivity of the sensor and increasing the electron transfer [47,48,49]. Interestingly, aptamers can be immobilised on the surface of graphene using pi-pi stacking, and graphene provides the sensor with excellent resistance to corrosion and heat [50,51,52,53]. The graphene/gold-hybrid form maximises the performance of the biosensor due to the easy aptamer immobilisation of graphene and excellent optical or electrical properties of the gold metal [54]. In addition, polymer/metal hybrids enable various designs of aptamer biosensors since the substrate can easily be deformed, and it is easy to obtain data on the neurotransmitter in real time due to the characteristics of the aptamer [55]. Therefore, aptamer-based sensors are often used as preliminary diagnostic tools because they enable high-throughput sensing with short detection times and easy immobilisation.

In this review, aptamer-based sensors are classified into five categories depending on the material used to detect the neurotransmitter: (1) gold-based, (2) gold/metal-hybrid-based, (3) graphene-based, (4) graphene/gold-hybrid-based, and (5) polymer/metal-hybrid-based (Table 1). Accordingly, various aptamer-based sensors that can rapidly and sensitively detect neurotransmitters are discussed (Figure 1C).

## 2. Gold-Based Aptamer Sensors

Gold nanoparticles (GNPs) can be stably synthesised via simple methods. Additionally, they are biocompatible and have excellent electrical conductivity, high surface-to-volume ratios, and unique optical properties, making them suitable for use in biosensors [73,74]. Furthermore, GNPs can easily be conjugated to aptamers via thiolation [75]. Because of these properties, various forms of gold-based aptamer sensors have been developed.

Recently, a GNP-based aptamer has successfully been used to detect serotonin (5-HT) by virtue of the optical characteristics of the GNPs [56]. Gold ions were reduced by ascorbic acid to synthesise the GNPs, which were then conjugated to the aptamer via electrostatic tuning of the positive charge on the GNP surface and the negative charge on the aptamer backbone. The aptamer was detached from the GNPs via the 5-HT reaction, and the GNPs were then aggregated via chloride ion loading. The solution colour change due to GNP aggregation was quantitated by UV/vis wavelength shifting. The limit of detection (LOD) was calculated at 1 ng/mL, and the selectivity was demonstrated by detecting 5-HT in human serum samples. Excellent repeatability and reproducibility were also confirmed. This study suggests that the GNP–aptamer sensor is suitable for detection of 5-HT.

In studies, GNPs have been synthesised in a variety of forms, which have then benefitted from testing by a variety of analytical methods [76]. For example, a study used aptamer-functionalised gold nanoflowers (Apt-GNFs) to successfully detect dopamine (DA) [57]. To construct this Apt-GNF, a 13-nm core GNP was first synthesised, then an aptamer was conjugated to the core GNP through a thiol group, and finally the GNP was allowed to grow along with the aptamer. LDI-MS was used for data analysis. A linear range of 28–140 nM was calculated, demonstrating excellent selectivity, reproducibility, and reproducibility. This approach is suitable for processes that require detection of trace amounts of neurotransmitters. 

**Figure 2:**
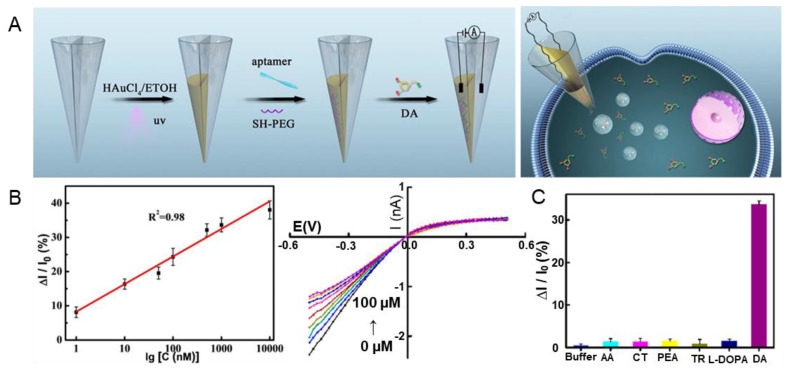
Gold-based dual-nanopore aptamer sensors for DA detection. (**A**) Schematic illustration of the fabrication process of a gold-based aptamer sensor. (**B**) Linear I-V curve indicating the linear range and LOD of DA, respectively. (**C**) Selectivity of the fabricated aptamer sensor. With permission from [58]. Copyright 2022, ACS. HAuCl_4_, Gold (III) chloride trihydrate; ETOH, ethanol; UV, ultraviolet; SH, thiol; PEG, polyethylene glycol; DA, dopamine; AA, ascorbic acid; CT, catechol; PEA, phenethylamine; TR, tyrosine; L-DOPA, 3,4-dihydroxyphenylalanine.

In addition to 3D nanostructures, aptamer sensors in the form of gold-film electrodes have been studied. In a study by Zhao et al., DA was successfully detected using a dual-nanopore aptamer sensor with a gold-film deposition [58]. In such dual nanopores, the inner space of the nanopipette is divided into two, and a gold film is deposited on one of the nanopores (Figure 2A). The dual-nanopore structure is fabricated using a laser pulling process. A thiolated aptamer that specifically binds to DA is attached to the gold-film-coated nanopore. The developed sensor successfully detected DA in a single pheochromocytoma (PC12) cell seeded in a microwell with a diameter of 100 μm and a height of 50 μm. The linear range and LOD were 1 nM–10 μM and 0.4 nM, respectively, based on a linear sweep voltammetry method (Figure 2B). Selectivity was also demonstrated by comparing the values from single HeLa and PC12 cells (Figure 2C). Excellent repeatability and reproducibility were also verified. It was reported that the nanopore sensor can be used up to five times. These results suggest that various types of sensors can potentially be fabricated using gold-based aptamer sensors.

The use of physically interdigitated gold-based electrodes and field-effect transistors (FETs) has been shown to be effective in the sensitive detection of analytes. In 2019, a group reported the detection of *Plasmodium falciparum* (pf) glutamate dehydrogenase in serum samples using an aptamer sensor in which FETs were structurally interlocked with gold electrodes [58]. The sensor was fabricated by connecting a gold electrode to the silicon oxide surface of the FET and immobilising the aptamer. Glutamate was detected in the linear range of 100 fM–10 nM using a buffer and serum as solvents, with calculated limits of detection (LOD) for glutamate of 16.7 and 48.6 pM, respectively. The sensor was also subjected to a selectivity test using interference molecules, such as human hydroxyglutarate dehydrogenase, human serum albumin, pf lactate dehydrogenase, and pf histidine-rich protein-II at 10 nM, demonstrating high selectivity suitable for actual sample analysis for malaria diagnosis. These findings suggest that this design could be implemented as a portable aptamer FET sensor in a point of care (POC) setting.

Gold nanostructures have unlimited potential depending on their application. They can be grown, attached by chemical bonding, or deposited onto the sensor surface. Similarly, the sensitivity of the sensor to detect an analyte can be fine-tuned. Modification of gold in the aptamer sensor improves the fundamental properties of the metal and aptamer binding performance, which is an important advantage of this biomaterial in that it does not damage the sensor.

## 3. Metal-Hybrid-Based Aptamer Sensors

Recently, various metals, such as Au, Pt, Ag, and Cu, have been used for biosensor development [77,78,79,80]. Various attempts have been made to use hybrid forms of metals to increase the sensitivity of biosensors. Bimetal nanoparticles are multiphase particles with novel characteristics due to the synergy between the metals and their role as catalysts while maintaining the advantages of each of the two separate metals [81].

**Figure 3:**
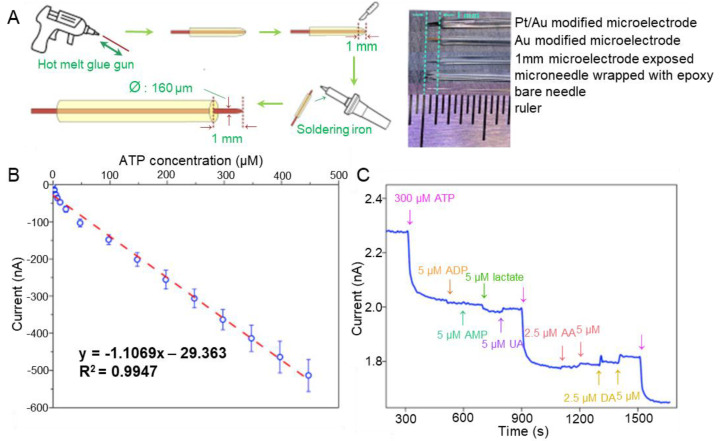
Au/Pt-hybrid-based needle-type aptamer sensors for detection of ATP as neurotransmitter. (**A**) Schematic illustration of the fabrication process of the metal-hybrid-based aptamer sensor. (**B**) Linear plot indicating the linear range and LOD of ATP. (**C**) Selectivity of the fabricated aptamer sensor. With permission from [61]. Copyright 2020, Elsevier. ATP, adenosine triphosphate; ADP, adenosine diphosphate; AMP, adenosine monophosphate; UA, uric acid; AA, ascorbic acid; DA, dopamine.

In 2022, a successful report was published on the detection of DA using an aptamer sensor based on a Ni/Pt hybrid [60]. An aptamer sensor was fabricated in a hybrid form, combining Ni and Pt as complementary metal oxide semiconductors (CMOS) to build a powerful biosensing substrate. The optimised sensor had a linear range of 10 fM–1000 fM. To test the reusability of the fabricated aptamer sensor, the authors removed the aptamer coating and conducted further functionalisation and biosensing measurement, thus verifying the reproducibility of the sensing properties of the aptamer sensor. These results suggest that bimetallic aptamer sensors are suitable for DA detection. Such bimetals can also be synthesised in various forms. In a recent study, a microelectrode biosensor based on 3D bimetal Au-Pt nanoflowers successfully detected adenosine triphosphate (ATP) via purinergic signalling [61]. The fabricated aptamer sensor was nanostructured layer by layer on a fixed steel acupuncture microneedle (Figure 3A)**.** Three-dimensional bimetallic Au/Pt nanoflowers were synthesised by anchoring a Au spike with improved conductivity, adding a catalytic layer of Pt on top, and finally adding the aptamer. The LOD and linear range were estimated at 2.5 μM and 2.5–447 μM, respectively (Figure 3B)**.** These results verified the good reproducibility of the fabricated aptamer sensor. ATP was successfully detected in the PC12 cell line, demonstrating the selectivity (Figure 3C)**.** Only negligible changes in current intensity were observed in the presence of interfering molecules. Moreover, repetitive addition of ATP significantly decreased the current response. These results suggest that bimetallic aptamer sensors are suitable for detecting neurotransmitters. Metal organic frameworks (MOFs), with large internal surface areas and various configurations and adjustable cavity sizes, were used as materials for catalysis [82,83]. Shi et al. succeeded in detecting DA by means of an aptamer sensor that used a cerium MOF-loaded silver nanocluster (MOFceAgNC) [62]. After the fabrication of cerium MOFs, MOFceAgNC was synthesised by adding Ag nanoclusters to the MOFs using a magnetic stirrer. MOFceAgNCs produce GNPs via a catalytic reaction with HAuCl_4_. The detection results were measured using SERS. The linear range and LOD were 0.01–0.25 nM and 0.008 nM, respectively. High selectivity was demonstrated by detecting DA in human serum samples, and excellent reproducibility was also confirmed. These results suggest that MOF-type metal-hybrid aptamer sensors are suitable for sensitive detection of DA.

Hybrid forms with two or more metals exhibit high sensitivity to analytes due to the synergistic effect of the metals. Moreover, hybridism circumvents the drawbacks of sensors that are based on a single metal and provides high selectivity. Combinations of metals in multiple phases may be particularly effective candidates for sensing a range of analytes, given that their sensing properties can vary depending on the metal types and proportions.

## 4. Graphene-Based Aptamer Sensors

Graphene is a carbon allotrope with a hexagonal honeycomb-shaped two-dimensional planar structure and has attracted much attention due to its excellent electrical conductivity, thermal conductivity, chemical and physical stability, and biocompatibility [84,85].

In 2021, Hwang et al. successfully detected DA at the fM level using crumpled graphene [63]. Using graphene-FET (G-FET), the researchers developed a sensor for DA detection. Since DA is electrically neutral, it cannot be detected via G-FET alone, which relies on electrical properties; thus, a polar aptamer was used as a detection probe and synthesised using pi-pi stacking. The linear range of the G-FET sensor was 2.5 aM–2.5 μM, and the LOD was 2.5 aM. The researchers reported that the LOD was 10 times lower than that of normal graphene. Excellent selectivity, reproducibility, and repeatability were also demonstrated. These results suggest that graphene-based aptamer sensors are suitable for monitoring neurotransmitters emitted from real cells.

There has also been an attempt to detect multiple neurotransmitters using graphene. Gao et al. successfully detected DA and 5-HT by arranging graphene with different aptamers on a single substrate in such a way that the circuits did not overlap [64]. After functionalisation of the graphene surface via electrochemical grafting methods, the aptamers that detect 5-HT and DA were subjected to the amine carboxylic acid reaction to increase their stability. In the case of 5-HT, when combined with an aptamer, the electrical response diminished as the distance between the aptamer and the graphene increases. In contrast, when the aptamer was combined with dopamine (DA), the electrical response became stronger as the aptamer moved closer to the graphene. The LODs and linear range were 10 pM and 10 pM–100 μM, respectively, for both 5-HT and DA. In addition, in the interference between substances, which is the key to multi-sensors, the response of the DA sensor when detecting 5-HT was 1.1%, and the response of the 5-HT sensor when detecting DA was −0.6%. The developed sensor was confirmed to have excellent selectivity, repeatability, and reproducibility while performing multiple monitoring of rat CSF 5-HT and DA. These study results suggest that a graphene-based aptamer sensor could potentially detect two or more neurotransmitters for complex disease monitoring. The biocompatibility of graphene and the sensitivity of the aforementioned graphene-based aptamer sensor showed the in vivo applicability of the sensor.

**Figure 4:**
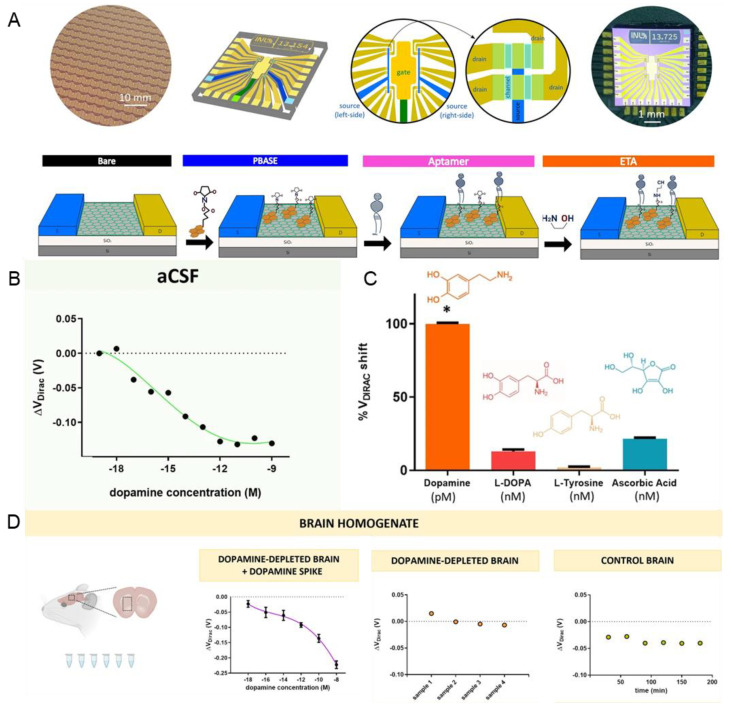
Graphene-based aptamer sensors for DA detection. (**A**) Schematic illustration of the fabrication process of graphene-based aptamer sensors. (**B**) Calibration plot indicating the linear range and LOD of DA. (**C**) Selectivity of the fabricated aptamer sensor. (**D**) Schematic illustration of the in vivo DA detection and the dot plots showing the in vivo detection performance. With permission from [65]. Copyright 2022, BMC. PBASE, pyrene-derived crosslinker; ETA, ethanolamine; aCSF, artificial cerebrospinal fluid. * *p* < 0.05.

In 2022, a group succeeded in quantitating DA using a graphene multi-transistor array (gMTA) functionalised with a selective DNA aptamer [65]. Micron-sized electrolyte-gated field-effect graphene transistors (EG-gFETs) were functionalised by attaching DNA aptamers and incorporated into graphene multi-transistor arrays (gMTA) (Figure 4A). Each gMTA chip consisted of an array of 20 EG-gFETs with individual interconnecting lines and groups of 10 transistors sharing a common source with individual gold drain electrodes connected to two common gold source electrodes. The authors obtained a calibrated curve to detect the dopamine in artificial cerebrospinal fluid to verify the sensitivity of the fabricated aptamer sensor. The LOD of the fabricated sensor showed a high sensitivity of 1 aM and a linear range of 1 aM–100 µM (Figure 4B). The fabricated aptamer sensor was successfully tested for selectivity for DA in the presence of interference molecules (Figure 4C). Responses to monoamine neurotransmitters, such as serotonin and norepinephrine, have not been tested, but aptamers have been reported as having reduced affinity for these neurotransmitters. Furthermore, this graphene-based aptamer sensor succeeded in detecting DA in the brains of rats with Parkinson’s disease (Figure 4D). Lastly, it was reported that 15680 EG-gFETs and 784 gMTAs were synthesised with a yield of 80% to prove repeatability and reproducibility. These results suggest that graphene-based aptamer sensors are suitable for in vivo applications.

The above studies suggest that graphene-based aptamer sensors have various advantages over other neurotransmitter sensors. Graphene has proven to be an easily implementable substrate for aptamer immobilisation that does not induce damage to biological samples. Moreover, the facile handling and superior analyte adsorption properties of graphene are expected to advance the development of aptamer sensors based on this biomaterial.

## 5. Metal/Graphene-Hybrid-Based Aptamer Sensors

It has been reported that metals, such as Al, Cu, Au, and Mo, used in sensors have high biocompatibility and excellent electron transfer characteristics, enabling them to be used as electrochemical sensors [86,87,88]. In addition, noble metals tend to be preferred over other non-metal materials because they have excellent optical properties and can be developed as optical sensors [89,90]. However, recent studies have reported that metallic materials have a low effect on the adsorption of biomolecules, reducing the LOD [91]. In addition, sensors based only on graphene show excellent adsorption to the analyte, but the sensing performance is significantly lower than that of metal-based sensors [92,93]. To compensate for these limitations, several studies have recently developed metal/graphene-hybrid-based aptamer sensors.

Mahmoud et al. developed an aptamer sensor decorated with GNPs on carboxylated carbon nanotubes (CNT) and succeeded in sensing histamine (HA) sensitively and selectively [66]. To construct this sensor, the aptamer was cast on the surface of a glassy carbon electrode (GCE), and a thiolated aptamer was incubated on the GNPs. This hybrid-material sensor recorded for HA a linear range and LOD of 0.46–35 nmol/L and 0.15 nmol/L, respectively. In addition, the developed sensor demonstrated high selectivity in the standard solution, human plasma, and canned tuna, and excellent repeatability and reproducibility were also confirmed. These results show that metal/graphene-hybrid-based aptamer sensors can potentially be used in health monitoring.

**Figure 5:**
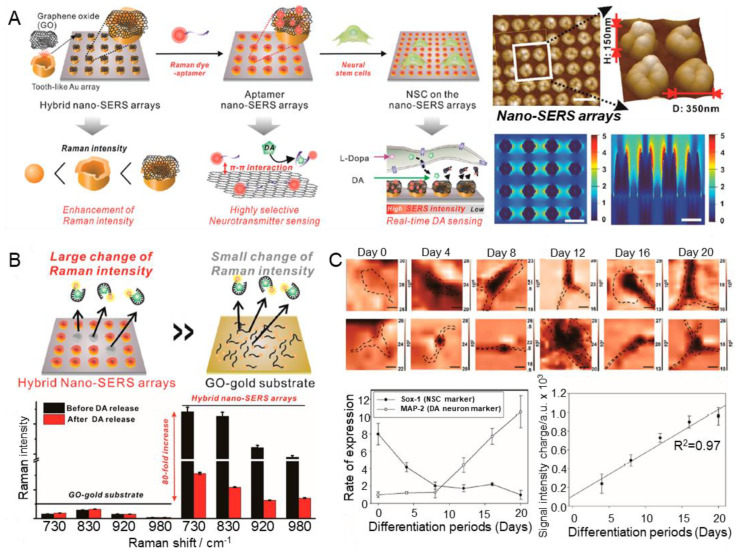
Metal/graphene-hybrid-based aptamer sensors for DA detection. (**A**) Schematic illustration of the fabrication process of metal/graphene-hybrid-based aptamer sensors. (**B**) Schematic illustration and plot indicating SERS signal change. (**C**) Raman mapping images by differentiation process and plot indicating the real-time DA-sensing performance. With permission from [68]. Copyright ACS. SERS, surface-enhanced Raman scattering; NSC, neural stem cell; DA, dopamine; GO, graphene oxide.

Reduced graphene oxide (rGO), Al, and Cu are widely used as electrochemical sensors because of their high chemical stability and ability to synergistically interact with various materials [94]. In one study, an aptamer platform based on a nanocomposite fused with CuAlO_2_ and rGO was developed to monitor DA [67]. rGO was homogenised through sonication, and the aptamer sensors were fabricated by stirring each metal material. The linear range of the fabricated aptamer sensor was 0.05 nM–10 μM, and the LOD was recorded as 0.017 nM. The author performed a selectivity test in a solution containing UA, AA, L-cysteine (L-cys), and glucose, and excellent sensing performance for DA was demonstrated. In addition, stability and reproducibility tests were successfully conducted for 30 days. This study suggests that the hybridisation of graphene and metal leads to higher stability and sensitivity than conventional aptamer capture probes. The high biocompatibility of gold and graphene is well known [95,96]. In addition, the uniform size and shape distribution of nanostructures can reduce signal variability; in particular, structural uniformity in surface-enhanced Raman scattering (SERS) can significantly amplify the analyte signal [97,98,99,100,101]. On the basis of these properties, Choi et al. developed a graphene/gold-hybrid SERS aptamer sensor that can detect DA by directly culturing cells on the sensor surface [68]. The high uniformity was achieved via laser interference lithography (LIL), the gold structure by electrochemical deposition, and the GO nanosheets by chemical conjugation (Figure 5A). The same authors confirmed the successful synthesis of the gold nanoarray structure of the tooth-like structure using AFM. In addition, they reported that precise nanostructure control can be achieved via a large-scale pattern area without a mask using LIL-based manufacturing. The fabricated SERS aptamer sensor exhibited sensitive sensing performance with a linear range of 1 nM–100 μM for DA. Interestingly, the fabricated SERS aptamer sensor demonstrated higher sensing performance than the existing gold film (Figure 5B). In particular, culture at the level of a single neural stem cell directly on the sensor demonstrated physical stability and real-time DA-sensing performance (Figure 5C). As expected, the authors reported that Raman signals tended to decrease in the Raman-mapping images of differentiated cells, whereas signal differences from undifferentiated NSCs gradually increased. These results demonstrate the highly advanced neurotransmitter detection properties of this type of aptamer sensor.

Metal/graphene hybrids can improve the signal between aptamers and analytes. Additionally, the properties of graphene can be exploited to use biosensors with immunoassays for simpler adsorption of metals or aptamers. This type of aptamer sensor, combining the advantages of metals and graphene, has efficient sensing performance. Moreover, it has better sensing performance than sensors that are based solely on graphene or metal. Nevertheless, additional research on the hybrid technology and the factors affecting detection performance is needed to achieve optimum performance.

## 6. Polymer/Metal-Hybrid-Based Aptamer Sensors

Since polymers are composed of repeatedly connected units, they can be fabricated so as to have flexible forms [102,103,104,105]. In addition, polymers have physical variables that determine their physical properties, and thus each polymer can be used in various ways [106,107]. Flexible devices have been reported to improve long-term cellular as well as in vivo monitoring by reducing tissue damage and immunological rejection [108,109]. In addition, a study has reported that the sensor is stable even after much bending or crumpling [110]. By virtue of these advantages, polymer/metal-hybrid-based aptamer sensors have been developed via positional and structural aptamer rearrangements that can be optimised for width and thickness, in such a way that extremely sensitive sensors can be made.

**Figure 6:**
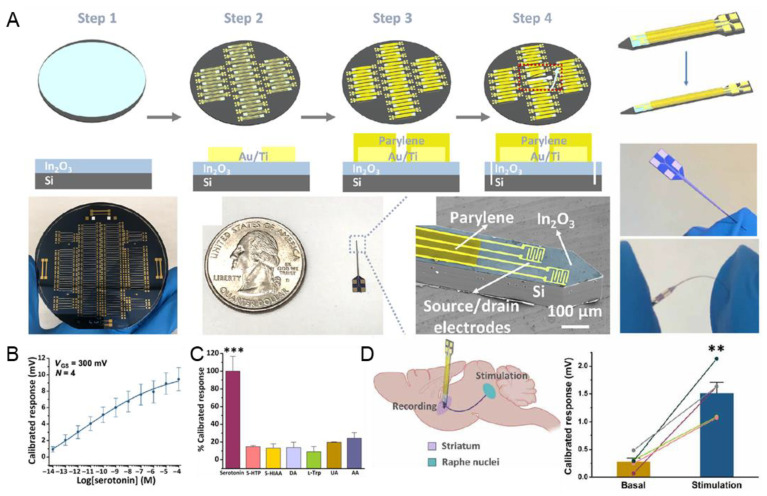
Polymer/metal-hybrid-based aptamer sensors for 5-HT detection. (**A**) Schematic illustration of the fabrication process of polymer/metal-hybrid-based aptamer sensors. (**B**) Linear plot indicating the linear range of 5-HT. (**C**) Selectivity of the fabricated aptamer sensor. (**D**) Schematic illustration of the in vivo 5-HT detection and the plot showing the detection performance. With permission from [69]. Copyright AAAS. L-5-HTP, L-5-hydroxytryptophan; 5-HIAA, 5-hydroxyindoleacetic acid; DA, dopamine; L-Trp, L-tryptophan; UA, uric acid; AA, ascorbic acid. ** *p* < 0.01, *** *p* < 0.001.

In 2021, a FET aptamer sensor using parylene and Au electrodes on a Si substrate was developed for highly sensitive and selective monitoring of 5-HT [69]. The authors modified In_2_O_3_ and Au/Ti electrodes through spin coating and oxygen plasma etching on a Si substrate (Figure 6A). The fabricated aptamer sensor detected a wide range of 5-HT concentrations, from 10 fM to 100 μM (Figure 6B). In addition, a selectivity test was conducted in the presence of several interference molecules, and high selectivity could be demonstrated by a sensitive response only to 5-HT (Figure 6C). Additionally, successful in vivo 5-HT sensing was performed by stimulating 5-HT secretion in the mouse brain (Figure 6D). In general, it has been reported that long-term sensing using Si-based sensors causes inflammation, but this study suggests high biocompatibility with free size control due to the high flexibility of the polymer base. Existing metal or silicon-based substrates are rigid, and the Young modulus is 200 GPa, but the Young modulus of brain tissue is <10 KPa [111]. A relatively high Young modulus causes tissue scarring, which leads to biosensor failure due to limited access between the sensing surface and the tissue interface [112]. To address these limitations of existing materials, flexible poly- (ether phthalate) (PET) and Au/Ti-hybrid aptamer sensors have recently been developed [70]. The authors fabricated an aptamer sensor for 5-HT by bonding a flexible neuro- from 10 fM to 100 M, for 5-HT in the artificial cerebrospinal fluid. In addition, high selectivity as an aptamer-using sensor was recorded for interfering molecules, such as DA, L-tryptophan, uric acid, and ascorbic acid. In addition, as a result of observing the time-dependent response to 5-HT, detection was possible in 10 min, showing a rapid sensing advantage due to the aptamer. This study suggests that, by adjusting the Young modulus, it is possible to circumvent histological and immunological problems and secure stability for biosensing.

It has been reported that real-time monitoring of neurotransmitters heterogeneously secreted in a cell can be realised via the immobilisation of two or more types of metal hybrids and specific aptamers [113]. On this basis, a polymer/metal-hybrid aptamer sensor in which a Au/Cr source drain is bonded to a polyimide body was developed [71]. The fabricated sensor was in the form of an FET, and the aptamer was immobilised by pi-pi stacking on a graphene film and assembled using a metal source-drain. The developed sensor had a wide linear range of 1 nM–10 μM and LOD of 10 pM for DA. In addition, it showed high selectivity for 5-HT, norepinephrine (NE), and GABA gamma-aminobutyric acid (GABA). Furthermore, the fabricated sensor had an excellent reversible response, even after DA was sensed. Accordingly, this study succeeded in developing an aptamer sensor capable of real-time monitoring of transient fluctuations in DA concentration at the cellular level.

Interestingly, the reported polymer/metal-hybrid-based aptamer sensor showed high signal recovery and sensor signal enhancement ability due to its material and structural characteristics. In particular, it was possible to detect neurotransmitters in real time through tissue-to-tissue access more easily than in the case of conventional rigid materials. Molecularly imprinted polymers (MIPs) are crosslinked polymers, which are synthesised based on the template molecule, and in which removal of the template generates the target-specific binding site [114]. Like other polymers, MIPs can be prepared rapidly and have high thermal, chemical, and mechanical stability. In addition, MIPs have the advantage of simple synthesis and of being low cost compared with other tailored recognition methods used in various processes [115]. A recent study has reported on dopamine detection via an MIP–aptamer sensor based on AuNPs and rGO electrodes [72]. The fabricated sensor utilised an aptamer and MIP double-detection system. The researchers formed a DA–aptamer complex by reacting with the DA and then conjugated the thiol group at the end of the aptamer to the surface of AuNPs. The MIP was created using a pyrrole polymer solution through cyclic voltammetry, and sulphuric acid was added to complete the MIP templates, from which dopamine was removed. The linear range was found to be 50 nmol/L–10 μmol/L, with an LOD of 47 nmol/L. The researchers observed that the MIP–aptamer double probe was more sensitive than the aptamer–AuNPs/rGO (LOD 93 nmol/L) and MIP–AuNPs/rGO (LOD 290 nmol/L). A selectivity test using ascorbic acid, uric acid, epinephrine, and catechol proved that the MIP–aptamer sensor reacted specifically to dopamine, suggesting higher selectivity than the aptamer–AuNPs/rGO and MIP–AuNPs/rGO. The sensor also demonstrated high reproducibility and repeatability, indicating that the aptamer sensor co-utilising MIP and aptamer is suitable for sensing a specific analyte in the presence of interfering molecules.

It has been proven that the combination of polymer and metal not only improves sensing functionalisation through flexible adsorption and penetration in vivo but also maximises the sensing performance of the aptamer sensor alone. A polymer/metal-based-hybrid aptamer sensor can be an excellent candidate to effectively detect various targets by taking advantage of the fact that results vary depending on how the material and composition are utilised.

## 7. Conclusions

This review summarised the recent research on aptamer-based biosensors for detecting and monitoring neurotransmitters in various materials. All the biomaterials reported above have demonstrated excellent efficacy for neurotransmitter detection. Metal-based aptamer sensors have excellent electron-transfer and signal-amplification properties, which vary according to the structural arrangement of the aptamer, and thus such sensors can be freely applied to electrochemical or optical systems. Graphene-based aptamer sensors feature excellent stability and high adsorption of analytes and chemicals. Likewise, the sensing performance of the metal/graphene hybrid is amplified by the synergistic effect of the strengths of each material. In addition, aptamer sensors based on polymer/metal hybrids can be used in various environments, and their high biocompatibility allows stable sensing in biological samples.

Since an increasing number of human nervous system diseases are being reported every year, researchers need to develop sensors that can easily detect various types of neurotransmitters. A particular problem is that in many situations the analyte of interest is present at low concentrations in a complex medium. Therefore, further research on the materials and technologies that can improve the sensitivity, accuracy, specificity, and reliability of biosensors is needed. In addition, although the sensing performance of biosensors with rigid or complex structures is usually stable and efficient, they are generally not suitable for real-time or sub-cellular monitoring. Therefore, in addition to increasing the sensing performance, efforts should be made to improve the biocompatibility and reproducibility of biosensors, and to prevent potential problems that may be experienced during on-site performance.

## Figures and Tables

**Table 1:** Materials used to construct aptamer sensors for neurotransmitters.

Material	Neurotransmitter	Detection Method	Linear Range	LOD	Ref.
Au	5-HT	LSPR	1–20 ng/mL	1 ng/mL	[56]
Au	DA	Mass spectrometry	20 μM–1.2 mM	-	[57]
Au	DA	Electrochemistry	1 nM–100 μM	0.4 nM	[58]
Au	Glutamate	FET	100 fM–10 nM	16.7, 48.6 pM	[59]
Ni, Pt	DA	Electronics	10 fM–1 pM	-	[60]
Au, pt	ATP	Electrochemistry	2.5–447 μM	2.5 μM	[61]
Cerium MOF, Ag	DA	SERS	10–250 μmol/L	8 pM	[62]
Graphene	DA	FET	2.5 aM–2.5 μM	2.5 aM	[63]
Graphene	DA, 5-HT	FET	10 pM–0.1 nM	10 pM, 10 pM	[64]
Graphene	DA	FET	10 aM–1 pM	1 aM	[65]
CNT, Au	HA	Electrochemistry	0.46–35 nmol/L	0.15 nM	[66]
rGO, CuAlO_2_	DA	Electrochemistry	50 pM–10 μM	17 pM	[67]
GO, Au	DA	SERS	10 nM–100 μM	-	[68]
Parylene, Au	5-HT	FET	10 fM–100 μM	-	[69]
PET, Au, Ti	5-HT	FET	1 pM–1 μM	-	[70]
Polyimide, Au, Cr	DA	Microtransistor	10 Fm–100 pM	10 pM	[71]
MIP, Au	DA	FET	50 nmol/L–10 μmol/L	47 nmol/L	[72]

FET: Field-effect transistor. SERS: Surface-enhanced Raman scattering. LSPR: Localised surface plasmon resonance.

## Data Availability

Not applicable.
